# Description of Sea Scurvy

**Published:** 1810-03

**Authors:** J. Howe

**Affiliations:** Assistant Surgeon of His Majesty's Ship Princess Carolina


### Description of Sea Scurvy.

By Mr. J. Howe, Assistant Surgeon of His Majesty's Ship Princess Carolina.

In the history of this disease, as it is met with on board of ships, it will be found to occur to seamen after living on food deficient of its nutritive properties. They will first complain of inaptitude to motion; shortness of breath on any exertion; debility. On examining the gums, they will be found to have an appearance very similar to that produced by mercury, unaccompanied with spitting; the breath fœtid. On examining the feet and ankles, slight œdema; the tunica conjunctiva shows the blood to be deficient in crassamentum by its want of colour. If the disease runs on, and the person has formerly had sores on his legs and feet, these places will become livid, and soon afterwards break out in the foulest ulcers. The system is now so enfeebled as not to be able to generate new flesh, to repair the injury; however, it may be stimulated until the general health of the system has been first repaired. Bleeding of the gums will, no doubt, sometimes occur before death; but this is only now and then met with in the most inveterate cases.

I saw a case of Scurvy in Guy's Hospital, of a man who had never been at sea; he appeared less debilitated; no œdema; and the blood less serous than in those cases which are met with at sea. The purple spots on the skin, in this case, were very numerous; and there was hæmorrhage rhage from the gums. Persons most predisposed to this disease, are those who have lately recovered from any other disorder, and where the digestive organs have suffered from whatever cause, as fatigue, cold, &c. No man is proof against the disease, although some will bear the exciting causes longer than others, without suffering in an equal degree.

The exciting cause is living upon food not sufficiently nourishing. This subject has been much canvassed, and some erroneous notions are still entertained on the subject. Our principal food is animal and vegetable. Animal food, under particular circumstances, such as the heat of warm climates, and from hot temperature of the summer solstice in Great Britain, soon becomes putrid; and the stomach loaths such food. In putrid meat there can be no nourishment. From the period of the death of the animal, there is a regular gradation takes place between the time that it is wholesome food and that of its putridity; the degrees between these extremes of animal food is regular, and according to the degree to which it approximates to putridity, it is less nourishing, and consequently more liable to produce scurvy. Some doubt has prevailed whether vegetable food could produce scurvy. Vegetable food is liable to decay, and in its decayed state it cannot nourish and support the system; consequently, the system will fall into disease, from a want of regular nourishment. It is not from putrid animal, or decayed vegetable food being taken into the stomach, from any sympathy or action it has upon the stomach, that scurvy is produced; it is from the want of food that is capable of nourishing the system, on which the lacteals are capable of acting, and fit for the purpose of being converted into blood, which will support the system. Cullen's definition is, "In cold climates occurring after living on putrescent salted animal food, with deficiency of recent vegetable matter; debility, bleeding of the gums, spots of different colours on the skin, for the most part livid, particularly at the roots of the hairs. By taking in such food, the frame becomes debilitated, and the lacteals lose part of their accustomed functions; consequently, may be said to fall into a state of disease. The indications of cure are to excite the lacteals to their accustomed functions, and restore the tone of the system. The acids are found to answer the first indication, by exciting the action of the lacteals; and of these, the citric acid very justly holds the first place. Nourishing food, and that of easy digestion, must be employed ployed to restore the tone of the system; as the whole of the primæ et secundæ viæ are in a torpid and very weak state."

I think it worthy of remark, that amongst the predisposing causes sometimes may be reckoned despondency, caused by tyranny and oppression; and it will be found in all these causes, the prognostic is less favorable. When men have suffered much from punishments, and fall into disease, their situation is more hopeless, and mortality will be much greater, not only in scurvy but in every other disease.

I have asserted, that in putrid animal food there is no nourishment. Although it has been admitted by some, that the human frame cannot be nourished by putrid animal food, yet it is said that some other animals have the power to change putrescent animal food into nourishing food. I recollect having heard a lecture, read in support of this doctrine: Two pigs had lived in the sewers of the streets of Paris for many months, and there became very fat. But I cannot persuade myself to believe, upon so slight a foundation, a thing so apparently contrary to the principles of nature. In the sewers of Paris, much, no doubt, would float, which the stomach of man would certainly loath, but still be very wholesome food for pigs, as potatoe-peelings, cabbage leaves, &c.; and that which has been too coarse for the delicate lacteals of man, may afterwards prove food for the stronger vessels of the swine; for it is not putrescent food only the lacteals will not take in.

The beef which is supplied to the sailors on board his Majesty's ships when at sea, is meat that has been dead generally twelve or more months; this has been kept from putrefaction by salt: but although this meat is not in the least putrescent, it nourishes not the system equally well with fresh meat; and it may be kept even from putrefaction, until it has lost all its nutritive qualities. According to the nutritive quality of the food, the system is preserved in strength, or falls into disease (scurvy.) The chief vegetable food on which sailors live, is flour and biscuit: what length of time flour will keep before it falls into so much decay that it will not support the system, I cannot say, but I know that a great deal of the flour, served to the men belonging to his Majesty's ships, is in a very decayed state, and is by no means equal in supporting the system in strength with fresh flour. The biscuit which is supplied to the men, is generally eight or more months old; old; in this state it has lost much of its nutritive quality, and supports the system not so well as new bread; after eight or ten months, bread falls very rapidly into decay.

Sugar is supplied in the quantity of six ounces per week to each man; there has been enough already printed on the subject of the nutritive property of this article, in strengthening and fattening the negroes in the West-Indies, to prove it possesses great nutritive properties. How long sugar will keep before it falls into a decayed state, I know not; but I have seen rotten sugar served out to a ship's company, when the ship has not been eight months absent from England. I do not conceive sufficient care is taken that all those articles which are served to his Majesty's ships, are purchased in that state which will best preserve their nutritive qualities. These articles, after they are sent to the victualler's stores, remain there until all the quantity of the same article in the store, which was on hand at the time, is sent away, and on board. That which has been longest on board is first to be used. All this I conceive to be very right, and mention it only to show the cause that the articles have been so long before they are issued to the men. The navy suffers much less now from this disease than formerly, in consequence of the judicious regulations of the commissioners for victualling his Majesty's navy; and I doubt not, still much more may and will be done for the sailor's advantage.

Why Cullen has made its occurrence in cold climates characteristic of the disease, I am unable to conjecture. The islands in the Pacific Ocean and the East Indies, are of a very warm temperature, where scurvy has brought so many seamen to an untimely grave; I much doubt if it does not oftener occur, and more die from this disease, in warm than in cold climates. It is not necessary that the food should either be salted or putrid, to produce the disease. Salt has no tendency to produce scurvy; and most of the beef on which those persons have been supported, which has produced the disease, has not been putrid; the salt has preserved it from putrefaction, but has not preserved all the nutritive properties of the beef entire.

Bleeding of the gums is considered one character; but many men have died of this disease where no hæmorrhage has taken place from the gums; nor is the skin always spotted. I shall leave nosologists to fix the proper character, and rather point out errors than correct them. The word scurvy has two objections; the one, that it is not expressive of the disease; and the other, that our ears are too often often annoyed with the application of scurvy to every trifling cutaneous affection;—a term expressive of deficient nourishment would be more proper.

*Portsmouth, February* 12, 1810.

*** We are very ready to find room for any articles by which we may contribute to the comfort of that valuable class of the community to whom we owe so much. Perhaps our Correspondent is mistaken in his opinion of putrid animal matter; but this is of less consequence, as such is rarely the food of sailors. The preservation of vegetable matter is much more important, and we have often wished for an opportunity of suggesting one simple improvement. Oatmeal is apt to become very soon sour in warm climates. As it is only, or principally, used for water-gruel, would it not be much better to use the whole grain or gritts as they are called? The French biscuit or rusks are also found to keep better than ours, and they have a mode of preserving the cabin store potted. Portable soups too, we conceive, might be given out by the captain or purser, to the men in health, at stated periods, and not merely reserved for sick store. During rain in warm weather, all the men might be taught to raise small sallad from raddish, mustard, or any other seeds, on wet flannel in plates.

### CRITICAL ANALYSIS

OF THE

